# Clinical Efficacy of Two Different Methods to Initiate Sensor-Augmented Insulin Pumps: A Randomized Controlled Trial

**DOI:** 10.1155/2016/4171789

**Published:** 2016-11-29

**Authors:** Jesus Moreno-Fernandez, Francisco Javier Gómez, Maria Ángeles Gálvez Moreno, Justo P. Castaño

**Affiliations:** ^1^Service of Endocrinology and Nutrition, Ciudad Real General University Hospital, SESCAM, Ciudad Real, Spain; ^2^Service of Endocrinology and Nutrition, La Mancha-Centro General Hospital, SESCAM, Alcázar de San Juan, Ciudad Real, Spain; ^3^Service of Endocrinology and Nutrition, Reina Sofia University Hospital, Maimónedes Institute of Biomedical Research of Córdoba (IMIBIC), Córdoba, Spain; ^4^Maimónedes Institute of Biomedical Research of Córdoba (IMIBIC), University of Córdoba, CIBERObn, Córdoba, Spain

## Abstract

*Aim*. To analyze clinical effect of a novel approach to initiate sensor-augmented insulin pumps in type 1 diabetes mellitus (T1DM) patients through early real-time continuous glucose monitoring (RT-CGM) initiation.* Methods*. A 26-week pilot study with T1DM subjects randomized (1 : 1) to start RT-CGM three weeks before continuous subcutaneous insulin infusion (CGM pre-CSII) or adding RT-CGM three weeks after continuous subcutaneous insulin infusion (CGM post-CSII).* Results*. Twenty-two patients were enrolled with a mean age of 36.6 yr. (range 19–59 yr.) and T1DM duration of 16.8 ± 10.6 yr. Higher adherence in CGM pre-CSII patients was confirmed at study end (84.6 ± 11.1% versus 64.0 ± 25.4%; *P* = 0.01). The two intervention groups had similar HbA_1c_ reduction at study end of −0.6% (*P* = 0.9). Hypoglycemic event frequency reduction was observed from baseline to study end only in CGM pre-CSII group (mean difference in change, −6.3%; 95% confidence interval, −12.0 to −0.5; *P* = 0.04). Moreover, no severe hypoglycemia was detected among CGM pre-CSII subjects during the study follow-up (0.0 ± 0.0 events versus 0.63 ± 1.0 events; *P* = 0.03). CGM pre-CSII patients showed better satisfaction than CGM post-CSII patients at the end of the study (27.3 ± 9.3 versus 32.9 ± 7.2; *P* = 0.04).* Conclusions*. CGM pre-CSII is a novel approach to improve glycemic control and satisfaction in type 1 diabetes sensor-augmented pump treated patients.

## 1. Introduction

Intensive therapy with the goal of maintaining tight glycemic control reduces diabetes chronic complications [1, 2]. Continuous subcutaneous insulin infusion (CSII) is an effective tool to improve type 1 diabetes mellitus (T1DM) control, although many patients remain with hemoglobin A1c (HbA_1c_) levels >7% [3–6]. Real-time continuous glucose monitoring (RT-CGM) can be added to CSII in order to improve glycemic control. Thus, T1DM patients usually start CSII and subsequently associate RT-CGM [7–10].

Low adherence to RT-CGM is the main limiting factor in most clinical studies [9–13]. Significant reductions in HbA_1c_ levels are usually achieved with at least 60–70% RT-CGM frequency of use [11–15]. Despite the possible beneficial effect expected or achieved, RT-CGM discontinuation due to sensor-related difficulties is a common problem in trials [9, 11–13, 16–19]. Real-life utilization of RT-CGM is even less in studies examining the decreasing potential beneficial results of these devices [8, 20].

Moreover, RT-CGM data interpretation can represent a challenge for sensor-augmented insulin pump treated patients. Early RT-CGM educational programs improve glycemic control in this group of patients [21]. In a previous study, we demonstrated that early RT-CGM introduction increases RT-CGM compliance and reduces time in hypoglycemia in sensor-augmented insulin pump treated patients [22].

Here, we have evaluated the final efficacy, safety, and satisfaction of this new model of starting dual device treatment.

## 2. Methods

### 2.1. Patients

Twenty-two CSII and RT-CGM naïve type 1 diabetes patients were finally enrolled at La Mancha-Centro Hospital and Ciudad Real University Hospital (Castilla-La Mancha Public Health Institute, SESCAM, Ciudad Real, Spain).

Inclusion criteria required ages between 14 and 65 years, T1DM diagnosed for >6 months, followed-up by the investigators for at least last 6 months, HbA_1c_ level of 7–9.5%, and treatment with basal/bolus multiple daily injections with rapid insulin analogs at mealtimes. Exclusion criteria were simultaneous participation in other studies, physical or intellectual limitations, unstabilized psychiatric disease in last 6 months, current or planned pregnancy, and breast-feeding. There were no exclusions for hypoglycemia unawareness, thyroid disease, or Addison's disease.

The protocol was approved by the reference Castilla-La Mancha Public Health Institute Ethic Committee. All participants, or parents in minors, provided written informed consent.

### 2.2. Study Treatment and Follow-Up

All patients wore six-day blinded continuous glucose monitoring sensors (CGMS® System Gold™; Medtronic Inc., Northridge, CA) to obtain baseline data after initial screening. At the first study visit, patients were randomly assigned (1 : 1) through sealed envelopes previously prepared by the Hospital Research Support Unit. Subjects were to receive RT-CGM during three weeks followed by CSII initiation (CGM pre-CSII) or starting with CSII and RT-CGM addition after three weeks (CGM post-CSII). CGM pre-CSII patients used capillary glucose levels, RT-CGM values, and glucose trends to modify their multiple daily insulin injection regimens for three weeks. Only capillary glucose levels were available by CGM post-CSII patients during the first three weeks. After the first three weeks all patients had complete dual devices prepared for diabetes treatment. No lower limit of RT-CGM wear was settled and both groups were equally encouraged to maximize RT-CGM use. Minimed Paradigm® Veo™ system (Medtronic Inc., Northridge, CA) and Optium Xceed® glucometer (Abbott Inc., Abbott Park, IL) were the electronic devices provided for the study. The same 4 h diabetes educational program, glucose targets, and scheduled contacts were settled for both groups. Workbooks and electronic information were gathered at all visits. Visits were conducted at 1, 2, 3, 4, 8, 12, and 26 weeks. Physical and laboratory exams were performed at baseline and at 12 and 26 weeks.

### 2.3. Efficacy and Security Assessments

The primary endpoint was RT-CMG frequency of use difference between both treatment groups. Adherence to RT-CGM was calculated as the amount of actual sensor use divided by expected sensor use of 100% per week. Secondary outcomes included (1) average change in HbA_1c_ levels between both groups; (2) average daily area under the curve (AUC) <70 mg/dL [3.9 mmol/L] and average daily AUC >180 mg/dL [10.0 mmol/L]; (3) incidence of severe hypoglycemic and hyperglycemic events; (4) quality of life (QoL); and (5) security.

Glycated hemoglobin was measured at Hospital Analysis Departments with the use of methods certified by National Glycohemoglobin Standardization Program. Average daily AUC <70 mg/dL [3.9 mmol/L] and average daily AUC >180 mg/dL [10.0 mmol/L] were measured by continuous glucose monitoring (CGM) data. Basal blinded CGM information was compared with end-of-study RT-CGM data to assess intervention effectiveness. Hypoglycemia events and severe hypoglycemia were defined in order to standardize concepts: an event of measured plasma or capillary glucose concentration ≤70 mg/dL and any hypoglycemia requiring assistance of another person to actively administer carbohydrates and glucagon or take other corrective actions were used, respectively [23]. Basal severe hypoglycemia frequency was calculated from 6-month previous period to study start. Reportable adverse events included severe hypoglycemia, hyperglycemia resulting in ketoacidosis, unexpected study-related or device-related events, and serious adverse events regardless of cause. These episodes were reported by subjects in their workbooks. We used CareLinkPro® software (Medtronic Inc, Northridge, CA) to download and interpret RT-CGM and pump use. Glucometers were also downloaded through this software with Optium Xceed USB Data Cable® (Abbott Inc, IL). Finally we assessed diabetes related QoL through the Spanish version of the standardized Diabetes Quality of Life Questionnaire (EsDQoL) at baseline and 26 weeks visits. EsDQoL include sections on “Satisfaction,” “Impact,” “Social/Vacational worry,” and “Diabetes related worry” with higher numbers indicating a poorer QoL.

### 2.4. Statistical Analysis

A between-group difference in the RT-CGM wear of 20% was chosen for the study. Eleven patients in each group had >80% power to detect a 20% difference between groups at the 0.05 significance level. An SD of 26.5% has been assumed. After solving initial financial limitations, we were able to include and complete follow-up of all twenty-two required patients.

Mann–Whitney *U* and Wilcoxon signed-rank nonparametric tests were used to analyze statistical differences between groups and differences between baseline and study end, respectively. Significance was taken at *P* < 0.05. Analyses were performed with IBM SPSS software version 12.0 for Windows (SPSS Inc., Chicago, IL).

## 3. Results

### 3.1. Patients

Eleven patients were finally randomized to each treatment group (CGM pre-CSII and CGM post-CSII). All subjects completed the study follow-up with a 100% completion across both groups. Demographics and baseline characteristics by intervention group are shown in Table 1.

### 3.2. RT-CGM Adherence

Higher frequency of RT-CGM use in CGM pre-CSII patients was detected at week 12 (87.2 ± 12.7% versus 67.9 ± 20.7%; *P* = 0.006). This greater RT-CGM adherence in CGM pre-CSII patients was confirmed at study end (84.6 ± 11.1% versus 64.0 ± 25.4%; *P* = 0.01). RT-CGM final compliance greater than 80% of the time was detected more often in CGM pre-CSII patients (72.7% versus 27.3%; *P* = 0.04).

### 3.3. Glycemic Control

Overall HbA_1c_ reduction with sensor-augmented insulin pumps was detected during follow-up (mean difference in change, −0.67%; 95% CI, −1.1, −0.1; *P* = 0.01). Both groups attained significant HbA_1c_ change from baseline ([CGM pre-CSII: mean difference in change, −0.63%; 95% CI, −1.18, −0.08; *P* = 0.04]; [CGM post-CSII: mean difference in change −0.56; 95% CI, −1.02, −0.11; *P* = 0.01]). The two intervention groups showed similar HbA_1c_ values at study end (7.0 ± 0.6 versus 7.1 ± 0.6; *P* = 0.9).

We corroborated an improvement in average daily AUC of <70 mg/dL in CGM pre-CSII patients at study end (0.7 ± 0.6 mg/dL/day versus 2.7 ± 2.5 mg/dL/day; *P* = 0.01). Furthermore, only CGM pre-CSII group showed a reduction in AUC of <70 mg/dL from baseline to the end of the study (mean difference in change, −1.8 mg/dL/day; 95% CI, −3.4, −0.2; *P* = 0.03).

A reduction in AUC of >180 mg/dL was detected in CGM pre-CSII patients (mean difference in change, −10.9 mg/dL/day; 95% CI, −3.4, −0.2; *P* = 0.03) whereas CGM post-CSII did not show this clinical benefit (mean difference in change, −11.1 mg/dL/day; 95% CI, −16.0, −0.2; *P* = 0.07). We did not find differences between groups in AUC of >180 mg/dL at study end (9.8 ± 7.1 mg/dL/day versus 8.4 ± 7.8 mg/dL/day, *P* > 0.05).

No differences in the rest of glycemic variables analyzed were found between groups (Table 2). Predictors of response were not detected in both groups of patients.

### 3.4. Hypoglycemia Events

A hypoglycemic event frequency reduction was observed from baseline to study end only in CGM pre-CSII group (mean difference in change, −6.3%; 95% CI, −12.0, −0.5; *P* = 0.04). All CGM pre-CSII patients showed less than 10% of capillary glycemic values <70 mg/dL (<3.9 mmol/L). Hypoglycemic event frequency was consistently high at study end among CGM post-CSII patients compared with CGM pre-CSII subjects (11.0 ± 8.1% versus 4.5 ± 3.2%; *P* = 0.03).

Severe hypoglycemia incidence was greater among CGM post-CSII subjects at study end (0.6 ± 1.0 events versus 0.0 ± 0.0 events; *P* = 0.03). Thus, no severe hypoglycemic episodes were detected or reported in the CGM pre-CSII group. CGM post-CSII patients reported severe hypoglycemia: one patient experienced three episodes due to persistent misconduct to hypoglycemia, one subject reported two severe hypoglycemia events, and two suffered one severe hypoglycemic episode each. Fifty percent of severe hypoglycemic events occurred during “off” RT-CGM period. Only one patient suffered one severe hypoglycemic event due to RT-CGM alert inattention. No between-group differences were detected during the study follow-up on automated insulin suspension rate use (CGM pre-CSII 53.3% versus CGM post-CSII 46.7%; *P* > 0.05) or low-glucose suspension threshold (CGM pre-CSII 53.6 ± 21.8 mg/dL versus CGM post-CSII 50.6 ± 11.5 mg/dL; *P* > 0.05).

### 3.5. Quality of Life

The use of sensor-augmented insulin pumps was associated with an overall improvement (reduction) in EsDQoL total scores (mean difference in change −11.0; 95% CI, −17.0, −5.1; *P* = 0.002). In addition, significant EsDQoL total and section score reductions were detected from baseline to the study end in the CGM pre-CSII group. CGM pre-CSII patients showed better satisfaction than CGM post-CSII patients at the study end (27.3 versus 32.9; *P* = 0.035). However, only social/vacational worry section of EsDQoL questionnaire improved in CGM post-CSII patients (see details in Table 3).

### 3.6. Safety

No episodes of device malfunction occurred. No patient died during the follow-up.

## 4. Discussion

Several studies have demonstrated the beneficial impact of sensor-augmented insulin pumps on glycemic control. At least 60–70% of RT-CGM adherence is required to attain glycemic control improvements, but many subjects discontinue from glucose sensors use, especially in long-term follow-up [8–20]. According to our previous study, RT-CGM initiation before CSII is associated with a significantly greater adherence to glucose sensors [22].

The novelty of our present results resides in hypoglycemia reduction associated with high frequency RT-CGM compliance. We demonstrated a significant decrease in hypoglycemic capillary levels and, most importantly, a severe hypoglycemic event reduction when sensor-augmented insulin pumps were early started with RT-CGM.

Sensor-augmented pump therapy with automated insulin suspension reduced the combined rate of severe (hypoglycemic seizure or coma) and moderate (hypoglycemia requiring assistance from another person) hypoglycemia in T1DM patients. These results were associated with a 68% RT-CGM adherence in the low-glucose suspension group [24]. In our study, approximately fifty percent of subjects in each treatment group had activated this function suggesting that severe hypoglycemia reduction may be achieved by alternative mechanisms related to RT-CGM use. Voluntary insulin suspensions and temporary changes in basal insulin infusion could have a role in this effect.

The use of sensor-augmented insulin pump therapy with the threshold-suspend feature has previously demonstrated a reducing nocturnal hypoglycemia effect without increasing HbA_1c_ values in patients with documented nocturnal hypoglycemia. In Bergenstal et al. report, nocturnal hypoglycemic events occurred less frequently (31.8%) in the threshold-suspend group than in the control group (1.5 ± 1.0 versus 2.2 ± 1.3 per patient-week, *P* < 0.001). Main inclusion criteria included wearing RT-CGM >80% of time during the run-in phase [25]. We detected a significant 6.3% reduction in total hypoglycemic capillary levels in CGM pre-CSII group with at least similar RT-CGM adherence. However, since CGM post-CSII patients did not achieve this clinical benefit, it seems conceivable that a higher RT-CGM compliance could be necessary to attain hypoglycemic frequency reduction.

The notion that hypoglycemia reduction can be achieved through early RT-CGM introduction is further substantiated by the fact that reduction in average daily AUC of <70 mg/dL was only detected in CGM pre-CSII patients. Previously, we reported between-group differences in final average daily AUC of <70 mg/dL, although we could not demonstrate intragroup reduction from baseline to the end of the study [22]. Increasing final study size could have augmented statistical power of previous findings.

Sensor-augmented insulin pump therapy was shown to decrease HbA_1c_ without a concomitant increase in hypoglycemia compared with MDI [18, 20]. RT-CGM adherence greater than 60–70% of the time is a necessary condition to attain this clinical benefit [9, 11–15]. Thus, each 10% increase in compliance is associated with a 41% increase in the probability of a 0.5% reduction in HbA_1c_ [12]. We detected an overall and intragroup HbA_1c_ reduction during the follow-up, although no between-group HbA_1c_ differences were detected at the study end. Despite a 20.6% increase in CGM pre-CSII group sensor wear, we did not find a greater HbA_1c_ reduction in these patients. CGM post-CSII subjects showed a RT-CGM adherence (64%) over the 60% threshold to start detecting HbA_1c_ improvements so, albeit limited, this adequate compliance may have masked between-group differences. Nevertheless, study size was not estimated to detect HbA_1c_ differences because the primary endpoint was RT-CGM frequency use.

Time spent in hyperglycemia was significantly shorter in sensor-augmented insulin pump-treated patients than with MDI or self-monitoring blood glucose [26]. Average daily AUC for glucose levels >180 mg/dL were lower when subjects activated their RT-CGM devices in sensor-augmented insulin pump treated patients; this effect was associated with a consistently high (81%) sensor adherence [15]. In our study, only CGM pre-CSII group showed a significant reduction in AUC of >180 mg/dL during the follow-up, although we did not detect between-group differences at the end of the study.

Our last set of analysis was aimed at assessing the influence of pre-CSII versus post-CSII on EsQoL. This revealed that CGM pre-CSII patients showed better satisfaction in EsQoL questionnaire. Indeed, all EsQoL scales improved in CGM pre-CSII group during the follow-up. To date, available studies have only provided weak and/or insufficient evidence to sustain that QoL improves with sensor-augmented insulin pump treatment [26]. The diversity of interventions applied and of ways to measure QoL likely makes it difficult to reach solid conclusions. Only the Eurythmics study with suboptimally controlled T1DM patients showed a reduction in QoL test scores with sensor-augmented pump treatment [27]. In our work, we compared two different methods of initiating dual device treatment and both intervention groups received after the first three weeks the same treatment. Therefore, clinical importance of QoL differences found herein can be considered both relevant and trustworthy.

There are, nevertheless, some limitations inherent to this study. First, these data correspond to a small sized trial, where the interventions were known to participants and investigators given the nature of medical devices. In addition, assessment of severe hypoglycemia relied on patient recall of episodes and workbooks, as official clinical register such as emergency assist records were not investigated to check patient reports. Actively enquiring about severe hypoglycemia in all visits was performed in order to avoid this limitation.

In conclusion, we suggest early RT-CGM introduction as a novel approach to behavioral management in sensor-augmented insulin pump treated patients. Maximizing RT-CGM compliance could provide additional glycemic control benefits beyond glycated hemoglobin.

## Figures and Tables

**Table 1:** Baseline characteristics of the patients.

	CGM pre-CSII	CGM post-CSII	Total
*N* (number)	11	11	22
Sex (female/male) (number)	6/5	5/6	11/11
Age (years) (mean, range)	38.5, 20–59	34.8, 19–45	36.6, 19–59
Body-mass index (Kg/m^2^) (mean ± SD)	25.7 ± 2.8	26.1 ± 3.6	25.9 ± 3.1
Diabetes duration (years) (mean ± SD)	18.6 ± 12.0	15.1 ± 9.1	16.8 ± 10.6
Daily insulin doses (units/Kg/day) (mean ± SD)	0.7 ± 0.4	0.8 ± 0.2	0.7 ± 0.3

RT-CGM pre-CSII: real-time continuous glucose monitoring before continuous subcutaneous insulin infusion; RT-CGM post-CSII: real-time continuous glucose monitoring after continuous subcutaneous insulin infusion.

**Table 2:** Glycemic outcomes.

	Baseline, mean ± SD			End of study, mean ± SD			Difference from baseline, MDC (95% CI, *P*)	
	CGM pre-CSII	CGM post-CSII	*P*	CGM pre-CSII	CGM post-CSII	*P*	CGM pre-CSII	CGM post-CSII
HbA_1c_, %	7.6 ± 0.4	7.6 ± 0.5	0.92	7.0 ± 0.6	7.1 ± 0.6	0.90	−0.63 (−1.18, −0.08; *P* = 0.04)	−0.56% (−1.02, −0.11; *P* = 0.01)
Average interstitial glucose, mg/dL	155 ± 21	157 ± 26	0.72	140 ± 21	130 ± 38	0.31	−14 (−24, −5; *P* = 0.01)	−25 (−48, −2; *P* = 0.03)
% capillary glucose levels <70 mg/dL	10.8 ± 9.7	15.5 ± 15.6	0.60	4.5 ± 3.2	11.0 ± 8.1	0.03	−6.3 (−12.0, −0.5; *P* = 0.04)	−4.5 (−7, 16; *P* = 0.45)
% capillary glucose levels >180 mg/dL	19.6 ± 16.3	28.6 ± 17.3	0.06	26.6 ± 13.1	40.0 ± 32.8	0.47	7.0 (−19.4, 5.4; *P* = 0.23)	11.4 (−18.0, 40.1; *P* = 0.36)
Average AUC <70 mg/dL/day	2.5 ± 2.3	2.5 ± 4.3	0.35	0.7 ± 0.6	2.7 ± 2.5	0.01	−1.8 (−3.4, −0.2; *P* = 0.03)	−2.8 (−3.5, 0.6; *P* = 0.19)
Average AUC >180 mg/dL/day	19.9 ± 10.6	20.0 ± 15.3	0.67	9.8 ± 7.1	8.4 ± 7.8	0.42	−10.9 (−3.4, −0.2; *P* = 0.03)	−11.1 (−16.0, −0.2; *P* = 0.07)
Severe hypoglycemia events	0.9 ± 1.1	1.5 ± 3.6	0.71	0.0 ± 0.0	0.6 ± 1.0	0.03	−0.9 (−1.7, −0.2; *P* = 0.04)	−0.9 (−2.8, 1.0; *P* = 0.32)

RT-CGM pre-CSII: real-time continuous glucose monitoring before continuous subcutaneous insulin infusion; RT-CGM post-CSII: real-time continuous glucose monitoring after continuous subcutaneous insulin infusion; MDC: mean difference in change; CI: confidence interval.

**Table 3:** Diabetes Quality of Life Questionnaire (EsDQoL).

	Baseline		End of study	
	CGM pre-CSII	CGM post-CSII	CGM pre-CSII	CGM post-CSII
Satisfaction	34.2 ± 9.9	33.4 ± 9.9	27.3 ± 9.3^*∗*^	32.9 ± 7.2
Impact	35.6 ± 8.7	33.6 ± 8.2	30.7 ± 6.5	31.0 ± 6.7
Social/Vacational worry	14.3 ± 5.6	14.0 ± 5.8	12.3 ± 4.0	11.3 ± 4.9
Diabetes related worry	11.0 ± 4.4	10.5 ± 4.1	9.9 ± 3.3	9.0 ± 3.4
Total	95.1 ± 23.1	91.4 ± 24.2	80.2 ± 18.3	84.2 ± 18.3

Values are presented as mean ± SD. EsDQoL: Diabetes Quality of Life Questionnaire (Spanish version); RT-CGM pre-CSII: real-time continuous glucose monitoring before continuous subcutaneous insulin infusion; RT-CGM post-CSII: real-time continuous glucose monitoring after continuous subcutaneous insulin infusion. ^*∗*^Between-groups differences, *P* < 0.05.
